# Decreased Lipid Profile and Oxidative Stress in Healthy Subjects Who Underwent Whole-Body Cryotherapy in Closed Cryochamber with Subsequent Kinesiotherapy

**DOI:** 10.1155/2019/7524878

**Published:** 2019-08-14

**Authors:** Agata Stanek, Ewa Romuk, Tomasz Wielkoszyński, Stanisław Bartuś, Grzegorz Cieślar, Armand Cholewka

**Affiliations:** ^1^Department of Internal Medicine, Angiology and Physical Medicine, School of Medicine with the Division of Dentistry in Zabrze, Medical University of Silesia, Batorego St. 15, 41-902 Bytom, Poland; ^2^Department of Biochemistry, School of Medicine with the Division of Dentistry in Zabrze, Medical University of Silesia, Jordana 19 St., 41-808 Zabrze, Poland; ^3^Higher School of Strategic Planning in Dąbrowa Górnicza, Kościelna 6 St., 41-300 Dąbrowa Górnicza, Poland; ^4^Second Department of Cardiology, Institute of Cardiology, Jagiellonian University Medical College, 17 Kopernika St., 31-501 Krakow, Poland; ^5^Department of Medical Physics, Chelkowski Institute of Physics, University of Silesia, 4 Uniwersytecka St., 40-007 Katowice, Poland

## Abstract

**Objective:**

The aim of the study was to estimate the impact of whole-body cryotherapy (WBC) and subsequent kinesiotherapy on oxidative stress and lipid profile when performed in a closed cryochamber on healthy subjects.

**Material and Methods:**

The effect of ten WBC procedures lasting 3 minutes a day followed by a 60-minute session kinesiotherapy on oxidative stress and lipid profile in healthy subjects (WBC group, *n* = 16) was investigated. The WBC group was compared to the kinesiotherapy only (KT; *n* = 16) group. The routine parameters of oxidative stress (antioxidant enzymatic and nonenzymatic antioxidant status, lipid peroxidation products, total oxidative status (TOS), and oxidative stress index (OSI)) and lipid profile were estimated one day before the beginning and one day after the completion of the research program.

**Results:**

After treatment, in the WBC group, a significant decrease of oxidative stress markers (TOS and OSI) and a significant increase of total antioxidant capacity were observed. The activity of plasma SOD-Mn and erythrocyte total SOD increased significantly in the WBC group. In the KT group, the erythrocyte activity of total SOD, CAT, and GR decreased significantly after the treatment. The levels of T-Chol and LDL-Chol decreased significantly after treatment in both groups, but the observed decrease of these lipid parameters in the WBC group was higher in comparison to the KT group. The level of TG decreased significantly after treatment in the WBC group only.

**Conclusion:**

WBC performed in a closed cryochamber followed by kinesiotherapy improves lipid profile and decreases oxidative stress in healthy subjects.

## 1. Introduction

Whole-body cryotherapy (WBC) is the therapeutic exposure of the total human body to extreme cold temperatures (below -100°C) for a short time (maximum up to 120-180 seconds) [1, 2]. Immediately after each cryotherapy procedure, subjects usually perform an appropriate set of kinesiotherapy exercises in order to increase and consolidate the beneficial effects of cryogenic temperature treatment [2].

The action of cryogenic temperatures causes several favorable physiological reactions such as an analgesic [1–3], anti-inflammatory [4–6], and the promotion of a circulatory effect [1, 2]. Cryogenic temperatures applied to the whole body also improve mood [7] and have a beneficial influence on the endocrine system [8, 9]. Additionally, the latest studies show that WBC may improve memory deficits [10], prooxidant-antioxidant balance [11, 12], lipid profile [13], and skin appearance [14, 15].

Until now, WBC has been used mainly in sports medicine [16, 17] and in the treatment of locomotor system diseases [18, 19]. Currently, WBC is used more and more frequently as a wellness method in healthy subjects, to help maintain good health [2, 20], but the mechanisms of WBC action are not fully known.

The effect of WBC treatment may depend on the type of cryochamber in which procedures are performed, the temperature and number of procedures, and whether WBC procedures are connected with a subsequent session of kinesiotherapy [21, 22]. In the available literature, there has been only one study [13], which estimated the influence of WBC on lipid profile parameters in healthy subjects, but WBC procedures were not connected with a subsequent session of kinesiotherapy, and the authors only estimated lipid profile parameters. Similarly, there have been a few studies, which showed a beneficial influence of WBC procedures on the prooxidant-antioxidant balance in healthy subjects, but they were performed in different circumstances (type of cryochamber, temperature, without subsequent sessions of kinesiotherapy), and very often, the authors only estimated the level of selected parameters of prooxidant-antioxidant balance. However, evaluation of oxidative stress severity must be comprised of the determination of oxidative stress markers as well as the activity of enzymatic and nonenzymatic antioxidant systems simultaneously [23, 24]. This is because disruption of the prooxidative-antioxidative balance may be caused by increased reactive oxygen species (ROS) production, impaired ROS elimination, or the combined effect of both processes [25].

In light of the above findings, the aim of this research was to assess the influence of WBC, performed in a closed cryochamber (Wroclawski type) with a subsequent session of kinesiotherapy, on oxidative stress and lipid profile simultaneously in healthy subjects. The scheme of whole-body cryotherapy procedures and estimation of oxidative stress parameters were similar to the methods used in our manuscript published earlier [21].

## 2. Materials and Methods

### 2.1. Subjects

The research protocol was approved by the Bioethical Committee of the Medical University of Silesia in Katowice (permission no. NN-6501-93/I/07), Poland. All analyzed subjects gave written consent for inclusion in the research. All investigations were conducted according to the Declaration of Helsinki principles (1964).

The research engaged 32 healthy nonsmoking male subjects who were divided randomly by a physician into two groups with an allocation ratio of 1 : 1 : 16 healthy subjects exposed to whole-body cryotherapy procedures with subsequent kinesiotherapy (WBC group, mean age 46.63 ± 1.5 years) and 16 healthy subjects exposed only to kinesiotherapy procedures (KT group, mean age 45.94 ± 1.24 years), with no significant difference in mean age and body mass index (BMI) between them.

All subjects were healthy volunteers who qualified for a routine complex treatment called cryorehabilitation including WBC (treated as an assisting component) with subsequent kinesiotherapy or kinesiotherapy procedures only, in order to promote and increase wellness. The subjects were not previously exposed to WBC.

Prior to the research, a resting electrocardiogram was performed in all the subjects and each subject was examined by a physician to exclude any coexisting diseases as well as any contraindications for WBC procedures. Before each session of WBC, the blood pressure was measured for each subject [2]. All the subjects were asked to avoid alcohol, any immunomodulators, immunostimulators, hormones, vitamins, minerals, or other substances with antioxidant properties and drugs for 4 weeks before and during the research. The diet of the subjects was not modified, although they were asked to abstain from the consumption of caffeine 12 hours prior to laboratory analyses. They also maintained the same mode of physical activity during the research. The demographic characteristics of the healthy subjects are shown in Table 1.

### 2.2. Scheme of Whole-Body Cryotherapy and Kinesiotherapy Procedures

The healthy subjects, depending on the group, were exposed either to a cycle of WBC procedures lasting 3 minutes a day with a subsequent 60-minute session of kinesiotherapy or to a 60-minute session of kinesiotherapy only for 10 consecutive days at the same time in the morning, excluding the weekend.

The WBC procedures were performed in a closed cryochamber (Wroclawski type) cooled by liquid nitrogen (produced by Creator, Poland). The cryochamber consisted of an antechamber with the temperature -60°C and a proper chamber, where the temperature reached -120°C. The subjects were let into the chamber in groups of four. Each entry to the cryochamber was preceded by a 30-second adaptation period in the antechamber. After adaptation to cryogenic temperatures in the antechamber, the subjects entered the proper chamber for 3 minutes. During the WBC procedure, all the subjects wore swimsuits, cotton socks, gloves, wooden shoes, and also dust masks to protect their mouths and noses and ear-protectors to cover their ears. The subjects were not allowed to wear jewelry, glasses, and contact lenses. Each subject was given information concerning the rules of behaviour in the cryochamber: the need for slow, shallow breathing and no touching each other while moving about (slow walking in circles) [20, 21, 26].

Directly after leaving the cryogenic chamber, the subjects underwent kinesiotherapy lasting for one hour. The program of kinesiotherapy was the same for all the subjects in both groups. The program of kinesiotherapy consisted of exercises on stationary bikes lasting 20 minutes (5 min.: 3.5 Rate of Perceived Exertion (RPE), 4 min.: 5 RPE, 2 min.: 7 RPE, 4 min.: 5 RPE, and 5 min.: 3.5 RPE), treadmill (20 minutes walking at 4.0 mph), and whole body stretching exercises lasting for 20 minutes. All the exercises were carried out under the supervision of physiotherapists [20]. All the subjects completed the research program, and no complications or side effects related to the WBC procedures were observed.

**Figure 1:**
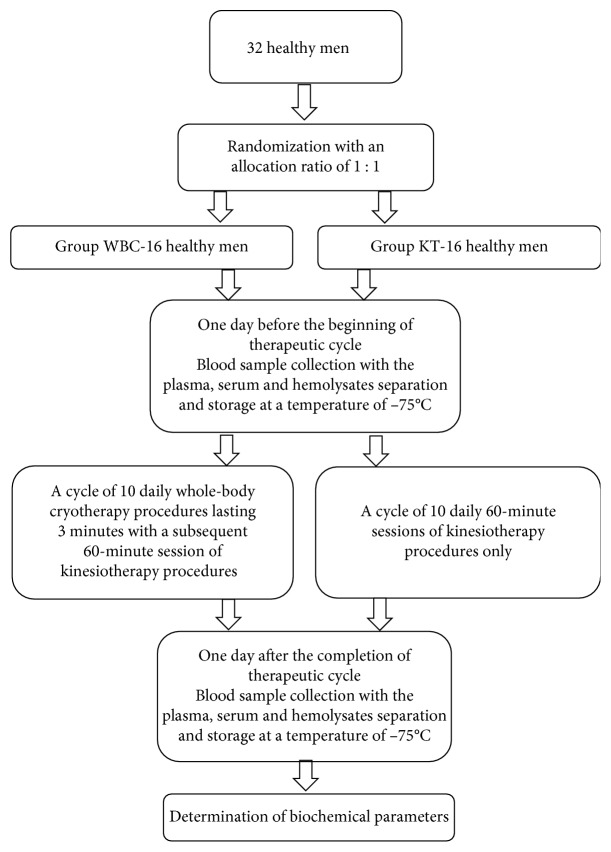
A scheme of the study protocol.

A scheme of the study protocol is presented in Figure 1.

### 2.3. Blood Sample Collection

Two blood samples of all the subjects were collected in the morning before the first meal: one on the day before the beginning of the procedure and the other on the day after completing the research program. Samples of whole blood (5 ml) were collected from the basilic vein into tubes containing ethylenediaminetetraacetic acid tripotassium salt (Sarstedt, S-Monovette with 1.6 mg/ml EDTA-K_3_) and into tubes with a clot activator (Sarstedt, S-Monovette). The blood samples were centrifuged (10 min., 900 g 4°C), and then, the plasma and serum were immediately separated and stored at a temperature of −75°C, until biochemical analyses could be performed. The red blood cells retained from the removal of EDTA-plasma were rinsed with an isotonic salt solution, and then, 10% of the hemolysates were prepared for further analyses. Hemoglobin concentration in the hemolysates was determined by the standard cyanmethemoglobin method. The inter- and intra-assay coefficients of variations (CV) were 1.1% and 2.4%, respectively.

### 2.4. Biochemical Analysis

#### 2.4.1. Determination of Lipid Profile Parameters

Total, HDL, and LDL cholesterol (T-Chol, HDL-Chol, LDL-Chol) and triglyceride (TG) concentrations in serum were estimated using routine techniques (Cobas Integra 400 plus analyzer, Roche Diagnostics, Mannheim, Germany). The concentrations were expressed in mg/dl. The inter- and intra-assay coefficients of variations (CV) were, respectively, 2.8% and 5.4% for T-Chol, 3.2% and 5.4% for HDL-Chol, 2.6% and 6.5% for LDL-Chol, and 2.5% and 7.6% for TG.

#### 2.4.2. Oxidative Stress Analysis

*(1) Determination of Lipid Peroxidation Products, Total Oxidative Status, and Oxidative Stress Index*. The intensity of plasma and the erythrocyte lipid peroxidation was measured spectrofluorimetrically as thiobarbituric acid-reactive substances (TBARS) according to Ohkawa et al. [27]. The TBARS concentrations were expressed as malondialdehyde (MDA) equivalents in *μ*mol/l in plasma or nmol/gHb in erythrocytes. The inter- and intra-assay coefficients of variations (CV) were 2.1% and 8.3%, respectively.

The total oxidant status (TOS) in serum was determined with the method described by Erel [28] and expressed in *μ*mol/l. The inter- and intra-assay coefficients of variations (CV) were 2.2% and 6.4%, respectively.

The oxidative stress index (OSI), an indicator of the degree of oxidative stress, was expressed as the ratio of total oxidant status (TOS) to total antioxidant capacity (FRAP) in arbitrary units [29].

*(2) Determination of Activity of Antioxidant Enzymes*. The activity of superoxide dismutase (SOD, E.C.1.15.1.1) in plasma and erythrocytes was measured by the Oyanagui method [30]. Enzymatic activity was expressed in nitrite unit (NU) in each mg of hemoglobin (Hb) or ml of blood plasma. One nitrite unit (1 NU) means a 50% inhibition of nitrite ion production by SOD in this method. SOD isoenzymes (SOD-Mn and SOD-ZnCu) were measured using potassium cyanide as the inhibitor of the SOD-ZnCu isoenzyme. The inter- and intra-assay coefficients of variations (CV) were 2.8% and 5.4%, respectively.

The erythrocyte catalase (CAT, E.C.1.11.1.6.) activity was assayed by the Aebi [31] kinetic method. It was expressed in IU/mgHb. The inter- and intra-assay coefficients of variations (CV) were 2.6% and 6.1%, respectively.

The activity of glutathione peroxidase (GPx, E.C.1.11.1.9.) in erythrocytes was determined by Paglia and Valentine's kinetic method [32], with t-butyl peroxide as a substrate and expressed as micromoles of NADPH oxidized per minute and normalized to one gram of hemoglobin (IU/gHb). The inter- and intra-assay coefficients of variations (CV) were 3.4% and 7.5%, respectively.

The erythrocyte glutathione reductase (GR, E.C.1.6.4.2) activity was determined by Richterich's kinetic method [33], expressed as micromoles of NADPH utilized per minute and normalized to one gram of hemoglobin (IU/gHb). The inter- and intra-assay coefficients of variations (CV) were 2.1% and 5.8%, respectively.

*(3) Determination of Nonenzymatic Antioxidant Status*. The total antioxidant capacity of plasma was assayed as the ferric reducing ability of plasma (FRAP) according to Benzie and Strain [34] and calibrated using Trolox. It was expressed in *μ*mol/l. The inter- and intra-assay coefficients of variations (CV) were 1.1% and 3.8%, respectively.

The concentration of protein sulfhydryl (PSH) was determined in serum by Koster's method [35], using dithionitrobenzoic acid (DTNB) and expressed in *μ*mol/l. The inter- and intra-assay coefficients of variations (CV) were 2.6% and 5.4%, respectively.

The concentration of uric acid (UA) in serum was measured by a uricase-peroxidase method [36] on the Cobas Integra 400 plus analyzer and expressed as mg/dl. The inter- and intra-assay coefficients of variations (CV) were 1.4% and 4.4%, respectively.

### 2.5. Statistical Analysis

For statistical analysis, the statistical package of Statistica 10 Pl software was used. For each parameter, the indicators of descriptive statistics were determined (mean value and standard deviation (SD)). The normality of the data distribution was checked using the Shapiro-Wilk test, while the homogeneity of the variance was checked by applying Levene's test. In order to compare differences between groups, either an independent sample Student *t* test was used or, alternatively, the Mann-Whitney *U* test. In the case of dependent samples, the Student *t* test was used or alternatively the Wilcoxon test. Differences at the significance level of *P* < 0.05 were considered as statistically significant.

## 3. Results

### 3.1. Lipid Profile Parameters

**Figure 2:**
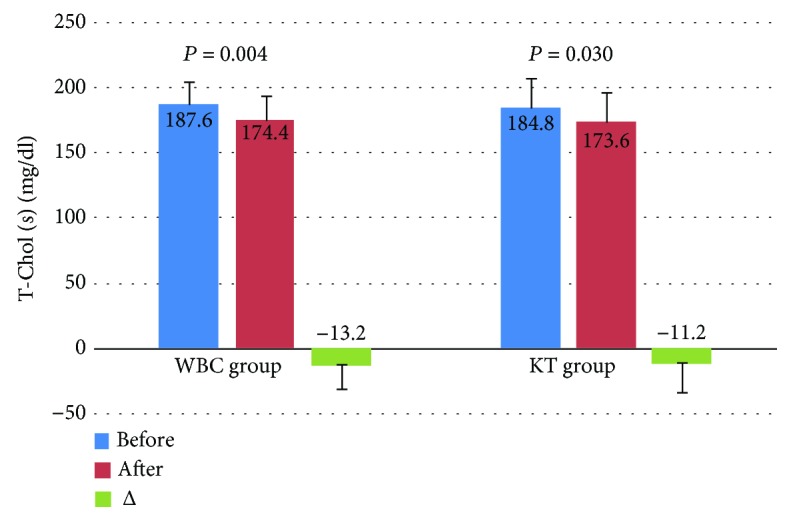
Levels of total cholesterol (T-Chol) (mean value ± standard deviation (SD)) in healthy subjects before and after the completion of a cycle of ten whole-body cryotherapy procedures with subsequent kinesiotherapy (WBC group) or a cycle of ten kinesiotherapy procedures only (KT group), with statistical analyses. (s): serum; *Δ*: difference prior to posttreatment.

**Figure 3:**
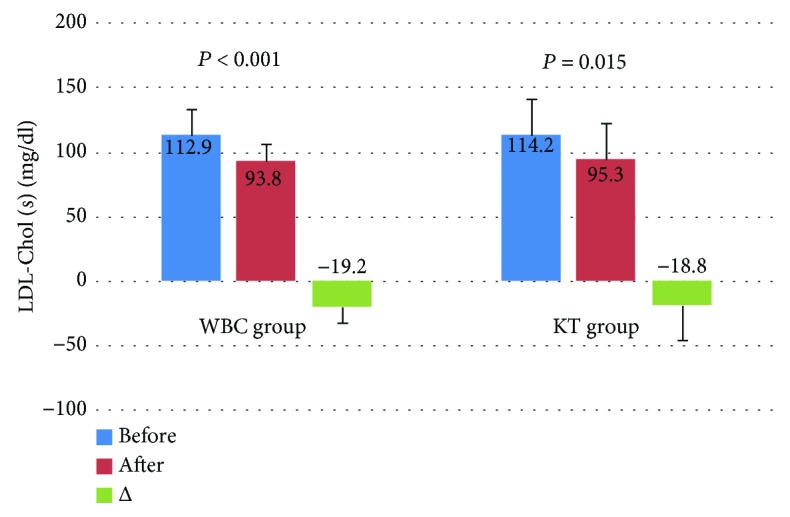
Levels of LDL cholesterol (LDL-Chol) (mean value ± standard deviation (SD)) in healthy subjects before and after the completion of a cycle of ten whole-body cryotherapy procedures with subsequent kinesiotherapy (WBC group) or a cycle of ten kinesiotherapy procedures only (KT group), with statistical analyses. (s): serum; *Δ*: difference prior to posttreatment.

**Figure 4:**
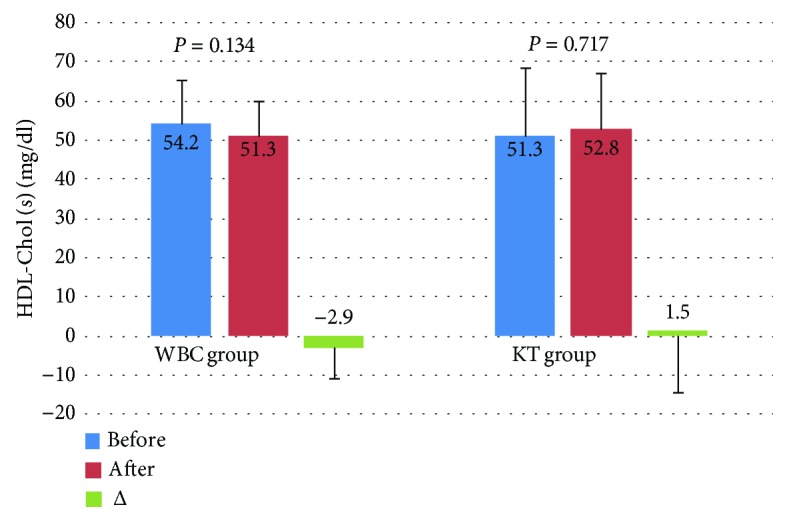
Levels of HDL cholesterol (HDL-Chol) (mean value ± standard deviation (SD)) in healthy subjects before and after the completion of a cycle of ten whole-body cryotherapy procedures with subsequent kinesiotherapy (WBC group) or a cycle of ten kinesiotherapy procedures only (KT group), with statistical analyses. (s): serum; *Δ*: difference prior to posttreatment.

**Figure 5:**
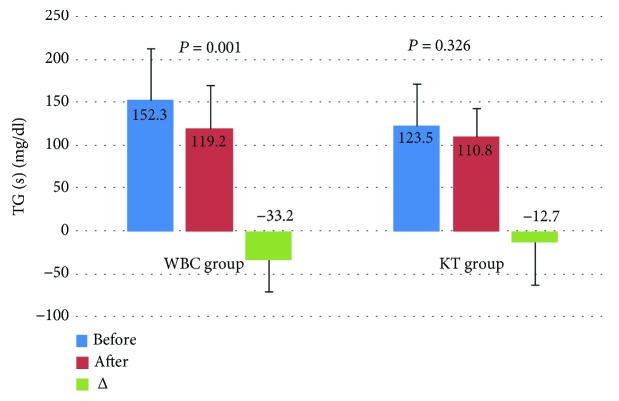
Levels of triglycerides (TG) (mean value ± standard deviation (SD)) in healthy subjects before and after the completion of a cycle of ten whole-body cryotherapy procedures with subsequent kinesiotherapy (WBC group) or a cycle of ten kinesiotherapy procedures only (KT group), with statistical analyses. (s): serum; *Δ*: difference prior to posttreatment.

Levels of T-Chol and LDL-Chol decreased significantly after the treatment in both groups, but the observed decrease of these lipid parameters in the WBC group was higher in comparison to the KT group. In turn, in the WBC group, the level of TG decreased significantly after the completion of the treatment in comparison to the KT group. The level of HDL-Chol did not change significantly in both groups (Figures 2–5).

### 3.2. Lipid Peroxidation Products, Total Oxidative Status, and Oxidative Stress Index

The subjects in the WBC group who underwent a ten-day long cycle of WBC procedures with subsequent kinesiotherapy had, after the completion of the treatment, a statistically significant decrease in plasma MDA, serum TOS, and value of OSI in comparison to initial values. The differences of these parameters prior to posttreatment values (*Δ*) in the WBC group were significantly higher in comparison to the KT group. The levels of MDA in erythrocyte did not change significantly in the WBC group. In the KT group of subjects who underwent a cycle of only kinesiotherapy, without previous cryotherapy procedures, no significant changes in the levels of plasma and erythrocyte MDA or serum TOS or OSI were observed after the completion of the treatment, in comparison to the initial values before the beginning of the kinesiotherapy cycle (Table 2).

### 3.3. Antioxidant Enzymes

The subjects in the WBC group had, after the completion of the treatment, a statistically significant increase in the plasma activity of SOD-Mn and erythrocyte total SOD.

However, the activity of plasma total SOD, SOD-CuZn, and erythrocyte activity of CAT, GPx, and GR did not change significantly in the WBC group after the treatment. In the KT group, the erythrocyte activity of total SOD, CAT, and GR, in contrast to the WBC group, decreased significantly after the treatment. Additionally, the difference prior to posttreatment values (*Δ*) of erythrocyte total SOD activity in the WBC group was significantly higher as compared to the KT group. Similarly, as in the WBC group, the activity of plasma total SOD, SOD-CuZn, and erythrocyte GPx did not change significantly in the KT group after the treatment.

Additionally, in the KT group, the activity of plasma SOD-Mn and SOD-CuZn did not change significantly after the treatment in comparison to the WBC group (Table 3).

### 3.4. Nonenzymatic Antioxidant Status

In the WBC group of subjects who underwent a ten-day long cycle of WBC procedures with subsequent kinesiotherapy, FRAP values increased significantly after the treatment. After the completion of the treatment, FRAP values were significantly higher in the WBC group when compared to the KT group. Similarly, the difference of this parameter prior to posttreatment values (*Δ*) in the WBC group was significantly higher in comparison to the KT group. The level of PSH and UA in the WBC group of subjects did not change significantly after the treatment. However, FRP values as well as PSH and UA concentration did not change significantly in the KT group of patients who underwent a cycle of kinesiotherapy only, without previous cryotherapy procedures (Table 4).

## 4. Discussion

Only a few papers have estimated the impact of WBC on lipid profile parameters. In experimental model animals (rats) who were exposed to WBC for 5 or 10 days, HDL and LDL cholesterol fraction decreased and total cholesterol levels in animals subjected to -60°C sessions for 10 days remained unchanged [37]. However, in the available literature so far, there has been only one paper on the effect of WBC on lipid profile in healthy subjects. Lubkowska et al. [13] reported reducing T-Ch, LDL-Ch, and TG levels and increasing HDL-Ch level after 20 sessions of WBC in healthy men, but after 10 sessions of WBC only, the LDL-Ch level decreased together with an observed HDL-Ch level increase in healthy men (cryogenic temperature -130°C, liquid nitrogen, without kinesiotherapy procedures). In another paper by these authors [38], in obese subjects without diet modification, a significant decrease in the level of LDL-Ch and TG was observed with a slight increase in the HDL-Ch level after WBC treatment (temperature -120°C, 2-3 minutes, the type of cryochamber not given), including two cryotherapy treatments of 20 daily sessions in the second and the last month of intervention. In our study, we observed a significant decrease in T-Chol, LDL-Ch, and TG levels after a ten-day long cycle of WBC procedures with subsequent kinesiotherapy in healthy subjects. The HDL-Ch level did not change significantly in the present study in the WBC group after the completion of the treatment.

So far, there have not been many reports on the impact of WBC procedures performed in different cryochambers on the prooxidant-antioxidant balance in healthy subjects.

In their study, Dugue et al. [39] observed a significant increase in the value of total peroxyl radical-trapping antioxidant capacity of plasma (TRAP) 2 minutes after cold stress in the first 4 weeks of their study. Thirty-five minutes after application of WBC, the values of TRAP did not vary from baseline values. But after 4 weeks, no changes in TRAP values after the WBC were noticed and no long-term changes in basal TRAP values were observed (temperature: -110°C, time: 2 minutes, coolant liquid nitrogen, 3 times per week for 12 weeks, without subsequent kinesiotherapy).

In the study of Lubkowska et al. [40], healthy men were exposed to a single WBC session (temperature: −130°C, time: 3 minutes, closed cryochamber, liquid nitrogen coolant) without subsequent kinesiotherapy. A significant increase in GPx and GR activities, with a simultaneous decrease in CAT and glutathione S-transferase activities, was observed. A significant increase in the concentration of glutathione, uric acid, albumins, and extraerythrocyte hemoglobin was also observed in the serum of the subjects. The level of TOS did not change after WBC procedure. The authors concluded that a single stimulation with cryogenic temperatures results in oxidative stress in a healthy body, but the level of this stress is not very high.

However, Mila-Kierzenkowska et al. [41] showed that even a single application of cryotherapy prior to exercise may have a beneficial impact on the antioxidant system of the body and alleviates signs of exercise-induced oxidative stress (a single session of WBC, temperature: -130°C, time: 1-2 min., cryochamber with cold retention, liquid air coolant).

In addition, Miller et al. [42] also observed an increase in total antioxidant status and the level of UA as a result of a series of short-term whole-body cryotherapy (10 WBC sessions, temperature: -130°C, time: 3 minutes, without subsequent kinesiotherapy) in healthy subjects.

In our research, the WBC group who underwent a ten-day long cycle of WBC procedures with subsequent kinesiotherapy had, after the completion of the treatment, a significant decrease in plasma MDA, serum TOS, and value of OSI. This is in comparison to initial values and the KT group of subjects who underwent a cycle of kinesiotherapy only, without previous cryotherapy procedures. Additionally, the subjects in the WBC group had, after the completion of the treatment, a significant increase in the plasma activity of SOD-Mn, FRAP values, and erythrocyte total SOD in comparison to the KT group. So, a ten-day long cycle of WBC procedures performed in a closed cryochamber cooled by liquid nitrogen at the temperature of -120°C with subsequent kinesiotherapy significantly decreased oxidative stress in healthy subjects.

The observed differences in the results of various researches may be related to the type of cryochamber being used and the coolant medium, in addition to the time and temperature of exposure to WBC as well as sex and body mass of subjects. Therefore, further studies are necessary [43].

### 4.1. Novelty of the Presented Research

In this research, for the first time, we assessed the influence of WBC, performed in a closed cryochamber (Wroclawski type) with a subsequent session of kinesiotherapy, on oxidative stress and lipid profile parameters, which were estimated simultaneously in healthy subjects.

There have been a few studies [41, 42], which showed a beneficial influence of WBC procedures on the prooxidant-antioxidant balance in healthy subjects, but they were performed in different circumstances (type of cryochamber, temperature, without subsequent sessions of kinesiotherapy), and very often, the authors only estimated the level of selected parameters of prooxidant-antioxidant balance. In the presented study, we estimated oxidative stress assessing the parameters of enzymatic and nonenzymatic antioxidant system as well as parameters of lipid peroxidation, total oxidant status, and oxidative stress index.

Similarly, in the available literature, there has been only one study [13], which estimated the influence of WBC on lipid profile parameters in healthy subjects, but WBC procedures were not connected with a subsequent session of kinesiotherapy, and the authors only estimated lipid profile parameters.

The obtained results in this presented study are similar to our previous research, in which a beneficial impact of ten WBC procedures performed in a cryochamber with cold retention followed by kinesiotherapy (temperature: -120°C, time: 3 min, liquid air coolant) on oxidative stress was observed in healthy subjects [20]. We also observed a similar influence of WBC procedures performed in closed cryochamber [21] as well as in cryochamber with cold retention [44] on oxidative stress in patients with ankylosing spondylitis. Additionally, we observed in patients with ankylosing spondylitis a significant decrease in T-Ch, LDL-Ch, and TG levels after the completion of ten daily procedures of WBC treatment (cryochamber with cold retention cooled by synthetic liquid air, temperature: -120°C) followed by kinesiotherapy [44].

To sum up, for the first time, in our studies, we showed that WBC performed in closed cryochamber and in cryochamber with cold retention has similar effect on oxidative stress and lipid profile. It also seems that it does not depend on the state of health. The obtained results give very important information about indications for the use of WBC by physicians and physiotherapists.

## 5. General Conclusion

Whole-body cryotherapy procedures performed in a closed cryochamber (Wroclawski type) followed by kinesiotherapy decrease lipid profile as well as oxidative stress in healthy subjects. Whole-body cryotherapy may be a beneficial wellness method.

## Figures and Tables

**Table 1:** Demographic characteristics of the research subjects.

Characteristic	WBC group (*n* = 16)	Kinesiotherapy group (*n* = 16)	*P* value
Age, years, mean (SD)	45.75 ± 2.08	46.06 ± 1.44	0.459
Sex (M/F)	16/0	16/0	—
BMI, kg/m^2^, mean (SD)	23.83 ± 4.5	24.26 ± 6.8	0.957
Smoking (yes/no)	0/16	0/16	—

SD: standard deviation; BMI: body mass index.

**Table 2:** Levels of lipid peroxidation parameters, total oxidative status (TOS), and oxidative stress index (OSI) (mean value ± standard deviation (SD)) in healthy subjects before and after the completion of a cycle of ten whole-body cryotherapy procedures with subsequent kinesiotherapy (WBC group) or a cycle of ten kinesiotherapy procedures only (KT group), with statistical analyses.

Parameters		WBC group	KT group	P
MDA (p) (*μ*mol/l)	Before	2.08 ± 0.47	2.30 ± 0.52	0.227
	After	1.63 ± 0.35	2.39 ± 0.52	**<0.001**
	*P* ^∗^	**0.013**	0.569	
	*Δ*	−0.45 ± 0.61	0.09 ± 0.70	**0.027**

MDA (e) (nmol/gHb)	Before	0.13 ± 0.01	0.17 ± 0.03	**<0.001**
	After	0.13 ± 0.01	0.17 ± 0.05	**0.010**
	*P* ^∗^	0.234	0.999	
	*Δ*	0.00 ± 0.01	0.00 ± 0.05	0.799

TOS (s) (*μ*mol/l)	Before	20.69 ± 4.99	10.12 ± 3.28	**<0.001**
	After	15.88 ± 3.77	11.97 ± 6.71	0.053
	*P* ^∗^	**0.006**	0.485	
	*Δ*	−4.81 ± 5.51	1.85 ± 7.80	**0.010**

OSI (p/s) (arbitrary unit)	Before	19.51 ± 7.75	6.84 ± 3.03	**<0.001**
	After	9.65 ± 3.18	10.79 ± 9.02	**0.637**
	*P* ^∗^	**<0.001**	0.196	
	*Δ*	−9.87 ± 7.50	3.96 ± 8.73	**<0.001**

*P*: statistical significance of differences between both groups of patients; *P*^∗^: statistical significance of differences between values before and after treatment in particular groups of patients. (p): plasma; (s): serum; (e): erythrocyte lysates; *Δ*: difference prior to posttreatment.

**Table 3:** Activities of antioxidant enzymes (mean value ± standard deviation (SD)) in healthy subjects before and after the completion of a cycle of ten whole-body cryotherapy procedures with subsequent kinesiotherapy (WBC group) or a cycle of ten kinesiotherapy procedures only (KT group), with statistical analyses.

Parameters		WBC group	KT group	*P*
Total SOD (p) (NU/ml)	Before	9.43 ± 2.77	11.2 ± 1.93	0.050
	After	9.40 ± 3.07	11.6 ± 3.26	0.062
	*P* ^∗^	0.818	0.679	
	*Δ*	−0.03 ± 4.74	0.40 ± 3.82	0.775

SOD-Mn (p) (NU/ml)	Before	3.96 ± 2.01	4.50 ± 1.41	0.387
	After	5.19 ± 2.05	5.16 ± 2.19	0.964
	*P* ^∗^	**0.030**	0.179	
	*Δ*	1.23 ± 1.96	0.65 ± 2.00	0.419

SOD-CuZn (p) (NU/ml)	Before	6.77 ± 2.70	6.72 ± 1.96	0.957
	After	4.84 ± 2.05	6.60 ± 2.92	0.060
	*P* ^∗^	0.063	0.918	
	*Δ*	−1.93 ± 4.05	−0.12 ± 3.05	0.166

Total SOD (e) (NU/mgHb)	Before	83.0 ± 9.82	114.0 ± 20.8	**<0.001**
	After	102.0 ± 13.1	98.5 ± 17.4	0.493
	*P* ^∗^	**0.001**	**0.002**	
	*Δ*	19.3 ± 13.9	−15.3 ± 12.7	**<0.001**

CAT (e) (IU/mgHb)	Before	104.0 ± 15.0	128.0 ± 11.2	0.352
	After	112.0 ± 11.2	111.0 ± 15.6	0.242
	*P* ^∗^	0.179	**0.049**	
	*Δ*	34.4 ± 87.0	39.9 ± 80.0	0.853

GPx (e) (IU/gHb)	Before	23.1 ± 3.86	23.8 ± 7.20	0.752
	After	22.0 ± 4.57	21.2 ± 3.71	0.606
	*P* ^∗^	0.121	0.179	
	*Δ*	−1.17 ± 3.31	−2.59 ± 7.99	0.519

GR (e) (IU/gHb)	Before	1.17 ± 0.37	1.49 ± 0.53	0.056
	After	0.99 ± 0.48	1.14 ± 0.61	0.450
	*P* ^∗^	0.134	**0.010**	
	*Δ*	−0.18 ± 0.43	−0.35 ± 0.44	0.267

*P*: statistical significance of differences between both groups of patients; *P*^∗^: statistical significance of differences between values before and after treatment in particular groups of subjects. (p): plasma; (e): erythrocyte lysates; *Δ*: difference prior to posttreatment.

**Table 4:** Levels of nonenzymatic antioxidants (mean value ± standard deviation (SD)) in healthy subjects before and after the completion of a cycle of ten whole-body cryotherapy procedures with subsequent kinesiotherapy (WBC group) or a cycle of ten kinesiotherapy procedures only (KT group), with statistical analyses.

Parameters		WBC group	KT group	*P*
FRAP (*μ*mol/l)	Before	646.1 ± 146.0	639.4 ± 83.8	0.876
	After	727.1 ± 114.4	609.9 ± 90.4	**0.003**
	*P* ^∗^	**<0.001**	0.088	
	*Δ*	81.0 ± 59.6	−29.5 ± 78.9	**<0.001**

PSH (s) (*μ*mol/l)	Before	613.2 ± 323.1	481.4 ± 126.8	0.145
	After	588.6 ± 312.7	451.4 ± 137.0	0.123
	*P* ^∗^	0.438	0.087	
	*Δ*	−24.6 ± 170.1	−30.1 ± 163.2	0.926

UA (s) (mg/dl)	Before	5.33 ± 1.50	5.61 ± 0.86	0.513
	After	5.47 ± 1.26	5.31 ± 1.24	0.720
	*P* ^∗^	0.352	0.198	
	*Δ*	0.15 ± 0.85	−0.30 ± 0.88	0.154

*P*: statistical significance of differences between both groups of patients; *P*^∗^: statistical significance of differences between values before and after treatment in particular groups of patients. (p): plasma; (s): serum; *Δ*: difference prior to posttreatment.

## Data Availability

The data used to support the findings of this study are included within the article.
